# Association of gene polymorphisms in FBN1 and TGF-β signaling with the susceptibility and prognostic outcomes of Stanford type B aortic dissection

**DOI:** 10.1186/s12920-022-01213-z

**Published:** 2022-03-20

**Authors:** Ling Sun, Yafei Chang, Peipei Jiang, Yitong Ma, Qinghua Yuan, Xiang Ma

**Affiliations:** 1 Department of Cardiology, Yingshang People’s Hospital, Fuyang, China; 2 Faculty of Forensic Medicine, Zhongshan School of Medicine, Sun Yat-Sen University, Guangzhou, China; 3 Department of Geriatrics, The Fourth People’s Hospital of Urumqi City, Ürümqi, China; 4 Department of Cardiology, First Affiliated Hospital of Xinjiang Medical University, 137 Liyushan South Road, Ürümqi, 830054 China; 5 Department of Cardiology, The Seventh Affiliated Hospital of Sun Yat-Sen University, Shenzhen, China

**Keywords:** Aortic dissection, *FBN1*, *TGFB1*, *TGFB2*, SNP, GMDR

## Abstract

**Background:**

This study is aimed at investigating the association of *Fibrillin-1* (*FBN1*) and transforming growth factor β (TGF-β) signaling-related gene polymorphisms with the susceptibility of Stanford type B aortic dissection (AD) and its clinical prognostic outcomes.

**Methods:**

Five single-nucleotide polymorphism (SNPs) (*FBN1*rs 145233125, rs201170905, rs11070646, *TGFB1*rs1800469, and *TGFB2*rs900) were analyzed in patients with Stanford type B AD (164) and healthy controls (317). Gene–gene and gene–environment interactions were assessed by generalized multifactor dimensionality reduction. A 4-year follow-up was performed for all AD patients.

**Results:**

G carriers of *FBN1* rs201170905 and *TGFB1* rs1800469 have an increased risk of Stanford type B AD. The interaction of *FBN1*, *TGFB1*, *TGFB2* and environmental promoted to the increased risk of type B AD (cross-validation consistency = 10/10, *P* = 0.001). Dominant models of *FBN1*rs145233125 TC + CC genotype (P = 0.028), *FBN1* rs201170905 AG + GG (*P* = 0.047) and *TGFB1* rs1800469 AG + GG (*P* = 0.052) were associated with an increased risk of death of Stanford type B AD. The recessive model of *FBN1* rs145233125 CC genotype (*P* < 0.001), *FBN1*rs201170905 GG (*P* < 0.001), *TGFB1* rs1800469 AG + GG genotype (*P* = 0.011) was associated with an increased risk of recurrence of chest pain in Stanford type B AD.

**Conclusions:**

The interactions of gene–gene and gene–environment are related with the risk of Stanford type B AD. C carriers of rs145233125, G carriers of rs201170905 and G carriers of rs1800469 may be the poor clinical outcome indicators of mortality and recurrent chest pain in Stanford type B AD.

**Supplementary Information:**

The online version contains supplementary material available at 10.1186/s12920-022-01213-z.

## Introduction

Stanford type B aortic dissection (AD) is a rare but serious cardiovascular emergency [1, 2], mainly through the interaction of gene mutations and environmental factors [3, 4]. Dysregulation and destruction of the cellular and extracellular components of the aortic wall result in progressive smooth muscle cells (SMC) depletion, extracellular matrix (ECM) destruction, and inflammation, which are pathologic changes that commonly lead to AD and rupture [5, 6].

*Fibrillin-1* (*FBN1*) gene encodes for Fibrillin-1 with 47 epidermal growth factor -like domains and seven transforming growth factor β (TGF-β) binding protein -like domains [7, 8]. Fibrillin-1 aggregates through cell secretion to form microfibrils; the microfibrils are located at extremity of the elastin extensions, joining the SMCs to the elastin lamellae, which is a necessary component of the elastic fibers in the aortic wall [9–12]. Loss of fibrillin-1 changes SMC phenotype and induces ECM remodeling, leading to aortic aneurysm/dissection [13]. *FBN1* was first recorded as an associated gene with Marfan syndrome (MFS) [14–17]. Researches manifested that, patients with a pathogenic *FBN1* variation are at risk for developing Marfan-like syndromes such as serious cardiovascular, skeletal, and ophthalmologic complications [14, 18–20]. Some recent researches also indicated that variants of *FBN1* was strongly related to the developing of thoracic aortic aneurysm or dissection (TAAD) in addition to MFS [21]. However, only a relatively few researches on genetic polymorphism and clinical prognosis between *FBN1* and Stanford type B AD.

TGF-β has a critical and fundamental role in the maturation and function of SMCs and aortic development [13]. TGF-β superfamily consists of at least 40 structurally and functionally related cytokines that are involved in various biological processes including embryonic development, ECM formation, immune regulation and inflammation, etc. [22, 23]. TGF-β1 is the main effective isotype on the cardiovascular system [24]. TGF-β1 increased expression of TGF-β type I receptor (TGFBR1) mutations causing Loeys–Dietz syndrome (LDS) which includes aggressive and early onset of both aortic aneurysms and dissections [25, 26]. The decrease of TGF-β2 levels caused by *TGFB2* mutation is an initiating step in the pathogenic of thoracic aortic disease [27]. Loss-of-function mutations in the genes encoding TGF-β ligands receptors are associated with heritable TAAD [28–31].

In addition, *FBN-1*, serves as a regulator of TGF-β signaling, can bind to LTBP-1 and regulates the bioavailability of TGF-β [26, 32]. Fibrillin-1 deficiency alter the matrix sequestration of the latent TGF-β complex, leading to the uncontrolled release of active TGF-β from the ECM and enhanced TGF-β signaling [12, 33–35]. In Marfan syndrome, the combination of excessive TGF-β synthesis and the uncontrolled release of TGF-β from *FBN1* deficient ECM contributes to aortic destruction [5, 36]. All the above indicated that TGF-β signaling and its related genes are involved in the progression of aortic disease.

Therefore, *FBN1* and TGF-β pathway-related gene variations are participated in the arising of aortic diseases by affecting architecture and function of aortic ECM and VSMCs. However, evidences for the interaction between *FBN1* and TGF-β pathway-related genetic polymorphisms in Stanford type B AD remain lacking before the submission of this manuscript. Moreover, we have not searched the correlation reports about the SNP of *FBN1*, *TGFB1* or *TGFB2* and the poor clinical prognosis of patients with Stanford type B AD. Given that the above reasons, the present study aimed to further explore the association of *FBN1*, *TGFB1*, and *TGFB2* genetic polymorphisms, gene–gene, and gene–environment interaction with susceptibility and clinical outcome of Stanford type B AD.

## Methods

### Ethical approval of the study program

This study was approved by the Ethics Committee of First Affiliated Hospital of Xinjiang Medical University. All participants and legal guardians of deceased participants have agreed and signed the informed consent voluntarily. The survey was carried on according to the principles of the Declaration of Helsinki.

### Study subjects and sample collection

All subjects were selected from the First Affiliated Hospital of Xinjiang Medical University between 2013 and 2016. Briefly, we enrolled 481 participants (164 type B AD patients and 317 Control groups). Patients with Stanford type B aortic dissection confirmed by aortography or aortic CTA were recruited in the case group. Control subjects were recruited from the same hospital and patients who were admitted for reasons without aortic disease by aortography or aortic CTA. Patients with coronary artery disease, cardiomyopathy, the bicuspid aortic valve or any other known aortic diseases were excluded from the study.

### Laboratory testing

The information, including hypertension, diabetes, age, gender, total cholesterol, triglyceride, low-density lipoprotein cholesterol and high-density lipoprotein cholesterol, was measured by the clinical laboratory department of the First Affiliated Hospital of Xinjiang Medical University with a biochemical analyzer. The definition of hypertension was as follows: systolic blood pressure ≥ 140 mmHg and/or a diastolic blood pressure ≥ 90 mmHg of three consecutive measurements on different days, for both arms [37]. Diabetes mellitus was diagnosed when two consecutive measurements on plasma glucose level ≥ 11.1 mmol/L and/or fasting plasma glucose levels ≥ 7.0 mmol/L 2 h after meal [38]. Smoking was defined as declaring regular tobacco use in the last 6 month.

### Genotyping

We selected five tag SNPs by screening National Center for Biotechnology Information SNP database (http://www.ncbi.nlm.nih.gov/SNP) and Haploview 4.2 software. Five tag SNPs, as follows: *FBN1* rs145233125, rs201170905, rs11070646, *TGFB1* rs1800469, and *TGFB2* rs900. The cut-off of minor allele frequency was set as > 0.05, and linkage disequilibrium patterns with r^2^ were set as > 0.8. Blood samples were collected from all subjects. With the use of a DNA extraction kit developed by Beijing Biotech Co. Ltd, Genomic DNA was extracted from peripheral vein blood leukocytes. Genotyping of all SNPs were performed at CapitalBio Corporation (Beijing, China) with MassARRAY platform (Agena Bioscience, San Diego, CA). The primers for PCR amplification and extension were designed by the MassARRAY Assay Design 4.0 software. The steps of the PCR cycling program, SAP (shrimp alkaline phosphatase) degestion and extension were performed according to the manufacturer's protocol. Extension products were desalted and detected using matrixassisted laser desorption ionization time-of-flight (MALDI-TOF). Finally, the results were analyzed using TYPER 4.0 software (Agena Bioscience, San Diego, CA). Genotyping was performed using a blinded method, without knowing any clinical data of the patient, and some genotyping samples (10%) were repeated to monitor the quality of genotyping.

### Followed up

We conducted a 4-year clinical followed-up for case group. All follow-up results were acquired by telephone calls, outpatient records or readmission. The baseline demographic data, clinical and clinical endpoint events of the selected patients were recorded. The primary endpoint was death due to the recurrence of AD, and the secondary endpoint was hospitalization for chest pain recurrence.

### Statistical analysis

SPSS version 22.0 software (SPSS, Inc., Chicago, IL) was used to conduct all statistical analyses. The t-test was used to compare the measurement data (represented by mean ± SD) between the AD and control subjects. Hardy–Weinberg equilibrium (HWE) was analysed to calculate the frequency distribution of genotype and allele in case and control groups. Generalized multifactor dimensionality reduction (GMDR) was used to analyze gene–gene and gene–environment interactions [39]. The Kaplan–Meier method was adopted to analyze the association of gene polymorphisms with survival outcomes and chest pain recurrence. Multivariate unconditional logistic regression analysis was used to analyze traditional risk factors of Stanford type B AD. The statistical significance level *P* value was set as < 0.05.

## Results

### Population information

Analysis of the general message of two groups found that did not show any differences in hypertension, triglyceride, total cholesterol, and low-density lipoprotein cholesterol between the case and control groups (*P* > 0.05). However, significant differences were found that systolic blood pressure, diastolic blood pressure, BMI, white blood cell count, creatinine, uric acid, glucose, glycosylated serum protein, high density lipoprotein cholesterol, hypertension, diabetes, smoking and drinking were associated with Stanford type B AD susceptibility (*P* < 0.05) (Table 1).

**Table 1:** General characteristics between case and control subjects

Characteristics	Case (N = 164)	Control (N = 317)	*P*
Age (years)	51.47 ± 11.29	55.43 ± 10.04	< 0.001
Male (n, %)	136 (82.9)	185 (58.4)	< 0.001
SBP (mmHg)	154.68 ± 30.56	126 ± 17.46	< 0.001
DBP (mmHg)	87.99 ± 18.64	78.97 ± 26.58	< 0.001
BMI (kg/m^2^)	26.28 ± 4.67	25.29 ± 3.34	0.033
WBC (10^9/L)	11.68 ± 4.19	6.41 ± 1.88	< 0.001
Creatinine (umol/L)	92.79 ± 96.87	69.18 ± 18.35	0.002
Uric acid (umol/L)	333.23 ± 108.02	309.94 ± 87.56	0.018
Glucose (mmol/L)	7.19 ± 2.39	5.49 ± 1.86	< 0.001
GSP (mmol/L)	2 ± 0.37	2.28 ± 0.52	< 0.001
Triglyceride (mmol/L)	1.56 ± 0.85	1.56 ± 0.72	0.949
Total cholesterol (mmol/L)	4.2 ± 1.01	4.05 ± 0.97	0.113
HDL-C (mmol/L)	1.06 ± 0.49	1.14 ± 0.34	0.063
LDL-C (mmol/L)	2.61 ± 0.78	2.68 ± 1.05	0.418
Hypertension (n, %)	128 (78.0)	132 (41.6)	0.106
Diabetes (n, %)	9 (5.5)	31 (9.8)	< 0.001
Smoking (n, %)	99 (60.4)	76 (24.0)	< 0.001
Drinking (n, %)	82 (50.0)	52 (16.4)	< 0.001

SBP, systolic blood pressure; DBP, diastolic blood pressure; BMI, body mass index; WBC, white blood cell; BUN, blood urea nitrogen; GSP, Glycosylated serum protein; HDL-C, high density lipoprotein cholesterol; LDL-C, low density lipoprotein cholesterol

### Genotype and allele frequencies

The genotype and allele distribution characteristics of SNPs in the case and control group are shown in Table 2. The genotype distributions of five SNPs for both case and control participants followed the Hardy–Weinberg equilibrium. There were significant differences for the genotype frequencies of *FBN1* rs201170905 (*P* = 0.011), *TGFB1* rs1800469 (*P* = 0.037) and the allele frequencies of *FBN1*rs201170905 (*P* = 0.001), *TGFB1* rs1800469 (*P* = 0.042) in the case group and the control group. No significant differences were observed between the case and control groups to the genotype frequencies and allele frequencies of *FBN1* rs145233125, rs11070646, *TGFB2* rs900 (*P* > 0.05).

**Table 2:** Description for genotype and allele frequencies in case and control group

SNP	Genotype/allele	Case, n (%) (N = 164)	Control, n (%) (N = 317)	*P*
rs145233125	TT	136 (82.93)	268 (84.54)	0.711
	TC	23 (14.02)	43 (13.56)	
	CC	5 (3.05)	6 (1.89)	
	T	295 (89.94)	579 (91.32)	0.480
	C	33 (10.06)	55 (8.68)	
rs11070646	CC	119 (72.56)	247 (77.92)	0.142
	CG	38 (23.17)	65 (20.50)	
	GG	7 (4.27)	5 (1.58)	
	C	276 (84.15)	559 (88.17)	0.081
	G	52 (15.85)	75 (11.83)	
rs201170905	AA	71 (43.29)	176 (55.52)	0.011
	AG	63 (38.41)	123 (38.80)	
	GG	30 (18.29)	18 (5.68)	
	A	205 (62.50)	475 (74.92)	0.001
	G	123 (37.50)	159 (25.08)	
rs1800469	AA	30 (18.29)	92 (29.02)	0.037
	AG	88 (53.66)	146 (46.06)	
	GG	46 (28.05)	79 (24.92)	
	A	148 (45.12)	330 (52.05)	0.042
	G	180 (54.88)	304 (47.95)	
rs900	AA	16 (9.76)	28 (8.83)	0.641
	AT	68 (41.46)	120 (37.85)	
	TT	80 (48.78)	169 (53.31)	
	A	100 (30.49)	176 (27.76)	0.375
	T	228 (69.51)	458 (72.24)	

### Analysis of the association between genetic models and Stanford type B AD risk

We further assessed the association between genetic models and the risk of Stanford type B AD. *FBN1* rs201170905 additive model GG genotype (OR 1.900; 95% CI 1.308–2.761, *P* = 0.001), *TGFB1* rs1800469 additive model AG genotype (OR 1.209; 95% CI 1.049–1.393, *P* = 0.013) or GG genotype (OR 1.193; 95% CI 1.009–1.411, *P* = 0.038) were found to be the risk factors for Stanford type B AD. However, there were no difference between the case and control groups in genotypes of *FBN1* rs145233125, rs11070646, *TGFB2* rs900 (*P* > 0.05) (Table 3).

**Table 3:** Analysis of the association between genetic models and aortic dissection risk

SNP	Genetic model	Genotype	OR	95% CI	*P*
rs145233125	Dominant	(TC + CC)/TT	1.042	0.868–1.251	0.647
	Recessive	CC/(TT + TC)	1.213	0.705–2.089	0.421
	Additive	TT	1		
		TC	1.018	0.842–1.231	0.850
		CC	1.216	0.706–2.095	0.415
rs11070646	Dominant	(CG + GG)/CC	1.109	0.942–1.305	0.192
	Recessive	GG/(CC + CG)	1.597	0.815–3.128	0.073
	Additive	CC	1		
		CG	1.069	0.908–1.26	0.405
		GG	1.620	0.826–3.175	0.062
rs201170905	Dominant	(AG + GG)/AA	1.183	1.038–1.348	0.011
	Recessive	GG/(AA + AG)	1.841	1.271–2.668	0.001
	Additive	AA	1		
		AG	1.078	0.946–1.227	0.253
		GG	1.900	1.308–2.761	0.001
rs1800469	Dominant	(AG + GG)/AA	1.203	1.058–1.369	0.010
	Recessive	GG/(AA + AG)	1.058	0.908–1.232	0.458
	Additive	AA	1		
		AG	1.209	1.049–1.393	0.013
		GG	1.193	1.009–1.411	0.038
rs900	Dominant	(AT + TT)/AA	0.962	0.762–1.215	0.739
	Recessive	TT/(AA + AT)	0.936	0.822–1.065	0.313
	Additive	AA	1		
		AT	0.997	0.778–1.277	0.981
		TT	0.938	0.738–1.191	0.706

### Gene–gene and gene–environment interaction

GMDR was used to analyze the interaction of the SNPs. The three-factor interaction model of *FBN1* rs201170905, *TGFB1* rs1800469, and *TGFB2* rs900 were the optimal model, through the maximum CVC (10/10) after 1000 permutation tests, and the maximum values of sign test (10) and test balance precision (0.5977), *P* = 0.0010 (Table 4).

**Table 4:** Generalized multifactor dimensionality reduction analysis of gene–gene interactions and aortic dissection risk

Model	Training bal. acc	Testing bal. acc	Sign test (p)	CV consistency
FBN1rs201170905	0.5653	0.5189	5 (0.6230)	9/10
FBN1rs201170905, TGFB2rs900	0.5968	0.5149	7(0.1719)	5/10
FBN1rs201170905, TGFB1rs1800469, TGFB2rs900	0.6391	0.5977	10 (0.0010)	10/10
FBN1rs11070646, FBN1rs201170905, TGFB1rs1800469, TGFB2rs900	0.6572	0.5417	8 (0.0547)	8/10
FBN1rs145233125, FBN1rsl 1,070,646, FBN1rs201170905, TGFB1rs1800469, TGFB2rs900	0.6739	0.5395	7 (0.1719)	10/10

Then, we assessed the gene–environment interaction and Stanford type B AD risk by GMDR. The result shown that the seven-factor interaction model of *FBN1*rs11070646, *FBN1*rs201170905, *TGFB1*rs1800469, *TGFB2*rs900, BMI ≥ 24 kg/m^2^, smoking, drinking and hypertension were considered the best model, with the maximum CVC (10/10) after 1000 permutation tests, and the maximum values of sign test (10) and testing balance accuracy (0.7560), *P* = 0.0010 (Table 5).

**Table 5:** Generalized multifactor dimensionality reduction analysis of gene–environment interactions and aortic dissection risk

Model	Training bal. acc	Testing bal. acc	Sign test (p)	CV consistency
smoking	0.6920	0.6703	10 (0.0010)	9/10
BMI ≥ 24 kg/m^2^, smoking	0.7322	0.6907	10 (0.0010)	7/10
FBN1rs201170905, BMI ≥ 24 kg/m^2^, smoking	0.7597	0.6922	10 (0.0010)	6/10
FBN1rs201170905, BMI ≥ 24 kg/m^2^, smoking, hypertension	0.7876	0.6912	10 (0.0010)	4/10
FBN1rs201170905, TGFB1rs1800469, TGFB2rs900, BMI ≥ 24 kg/m^2^, smoking	0.8295	0.6798	10 (0.0010)	8/10
FBN1rs201170905, TGFB1rs1800469, TGFB2rs900, BMI ≥ 24 kg/m^2^, smoking, hypertension	0.8717	0.7639	10 (0 0010)	9/10
FBN1rs11070646, FBN1rs201170905, TGFB1rs1800469, TGFB2rs900, BMI ≥ 24 kg/m^2^, smoking, hypertension	0.9078	0.7380	10 (0.0010)	5/10
FBN1rs11070646, FBN1rs201170905, TGFB1rs1800469, TGFB2rs900, BMI ≥ 24 kg/m^2^, smoking, drinking, hypertension	0.9330	0.7560	10 (0.0010)	10/10
FBN1rs11070646, FBN1rs201170905, TGFB1rs1800469, TGFB2rs900, BMI ≥ 24 kg/m^2^, dyslipidemia, smoking, drinking, hypertension	0.9539	0.7367	10 (0.0010)	10/10

### Logistic regression analysis of risk factors for Stanford type B AD

Multivariate unconditional logistic regression analysis was used to analyze the data. The valuable and empirical variables were included in the multivariate unconditional logistic regression analysis (Additional file 1: Table S1), and the variables were introduced in the equation. Glucose, hypertension, diabetes, smoking, and drinking were the risk factors of Stanford type B AD after adjusting the factors such as age, gender. Especially in the Hypertension group, the risk of Stanford type B AD increased 4.586-fold (OR 4.586, 95% CI 2.627–8.006, *P* < 0.001) compared with normal population.

### Correlation of SNPS with mortality risk in Stanford type B AD patients

**Fig. 1:**
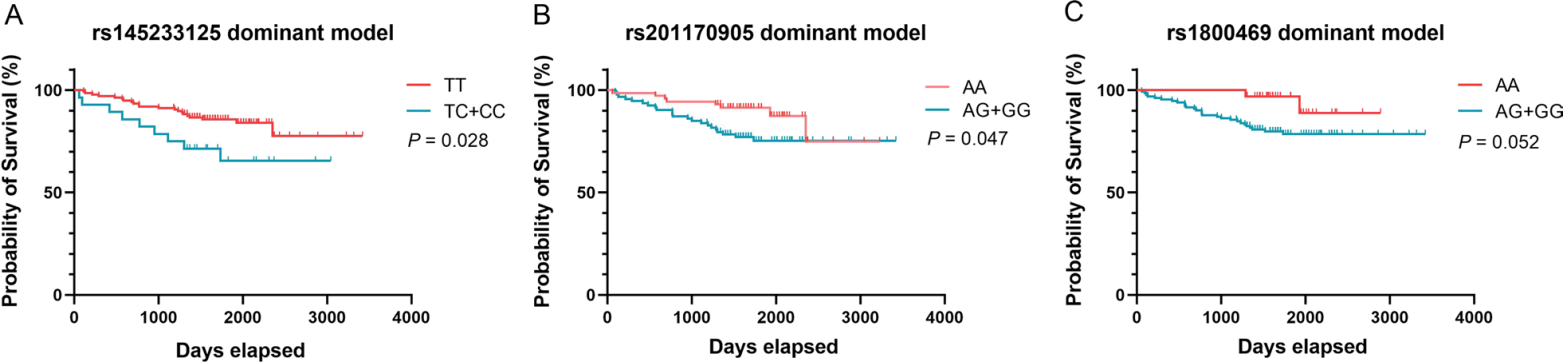
**a** Kaplan–Meier analysis of the overall survival based on FBN1 rs145233125 dominant model. **b** Kaplan–Meier analysis of the overall survival based on the FBN1 rs201170905 dominant model. **c** Kaplan–Meier analysis of the overall survival based on the TGFB1 rs11800469 dominant model

Among 317 patients included in the 4-year follow-up, 30 patients died due to the recurrence of Stanford type B AD. We use Kaplan–Meier method to analyze the association of tag SNPs and clinical outcomes in patients with Stanford type B AD. The results shown that there were no significant differences between the genetic models of *FBN1*rs11070646 and *TGFB2*rs900 and the risk of death (*P* > 0.05). However, dominant models of *FBN1*rs145233125 TC + CC genotype (*P* = 0.028), rs201170905 AG + GG genotype (*P* = 0.047) and *TGFB1*rs1800469 AG + GG genotype (of borderline statistical significance, *P* = 0.052) were associated with an increased mortality risk (Fig. 1).

### Correlation of SNPS with chest pain recurrence in Stanford type B AD patients

**Fig. 2:**
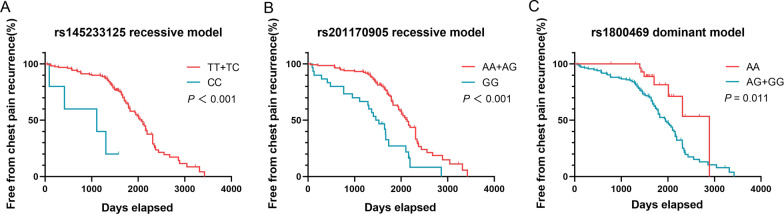
**a** Kaplan–Meier analysis of freedom from recurrence of chest pain based on the FBN1 rs145233125 recessive model. **b** Kaplan–Meier analysis of freedom from recurrence of chest pain based on the FBN1 rs201170905 recessive model. **c** Kaplan–Meier analysis of freedom from recurrence of chest pain based on the TGFB1 rs11800469 dominant model

Follow-up result shown 93 patients had recurrent chest pain in type B AD. The recessive models of *FBN1*rs145233125 CC genotype, rs201170905 GG genotype and the dominant model of *TGFB1*rs1800469 AG + GG genotype were found to be associated with an increased risk of recurrence of chest pain by Kaplan–Meier *(P* < 0.05*).*The association between mortality risk and other genetic models did not show any statistically significant differences (*P* > 0.05) (Fig. 2).

## Discussion

In our present study, we found that the genetic mutations in *FBN1* and TGF-β signaling, and environmental influences and conditions were associated with Stanford type B AD and adverse outcomes. This may be caused by the interaction of multi-genes and environment.

A previous study revealed that two single nucleotide polymorphisms (SNPs, rs2118181 and rs10519177) in the *FBN-1* gene were associated with thoracic aortic dissection (TAD), thoracic aortic aneurysm (TAA), and TAAD [8, 40]. Furthermore, another study has also demonstrated that carriers of the *FBN1*rs2118181 risk variant had greater risk for TAD [41]. However, before this, related research about rs201170905 and the susceptibility of AD has not been found. Our study confirmed that A allele carriers of the *FBN1* rs201170905 polymorphism was considered to the genetic influence factors of Stanford type B AD. Rs201170905 is located within the *FBN1* gene introns; Intronic variations is mainly through a selective shearing to influence protein sequence and function. Mutant fibrillin-1 initiates disease-causing changes in the extracellular matrix by decreasing the level of functional microfibrils and activating TGF-β signaling, leading to AD eventually [42].

The current study demonstrated that *TGFB1* gene variants were associated with Stanford type B AD. *TGFB1*, as a cytokine, participates in a broad range of cellular regulatory processes and associated with different kinds of diseases including aortic aneurysm [32]. Increased TGFβ1 levels are linked to MFS caused by *FBN1* mutations and subsequent defects in signaling system [43]. Previous studies reported that mutations in TGF-β signaling pathway-related genes cause syndromic TAAD, such as MFS and LDS [44]. Rs1800469 (T-509C) is a variation in the promoter region of the *TGFB1* gene that affects gene transcriptional activity [45]. Other research indicated the increased risk of abdominal aortic aneurysm for individuals with the *TGFB1* rs1800469 TT genotype compared with the CC genotype [46–50]. In the current study, dominant model of *TGFB1* rs1800469 have a higher risk of Stanford type B AD. By contrary, a previous study demonstrated that the recessive model and additive model of rs1800469, but not dominant model, were related to abdominal aortic aneurysm [45], which may be because of differences in the environment in which people live.

Stanford type B AD was bound up with genetic and environmental factors [51]. Therefore, we performed an GMDR analysis which manifested the interactions between *FBN1* rs201170905, *TGFB1* rs1800469, *TGFB2* rs900 and circumstance factors contributed to Stanford type B AD. One study has demonstrated that interactions between *TGFB1* gene polymorphism and environmental factors promoted abdominal aortic aneurysm [52]. *FBN-1,* serves as a regulator of TGF-β signaling, has been shown to interact with *TGFB* [53]. Frameshift mutations and nonsense mutations may lead to a decrease in FBN1 protein levels [54]. This reduce causes the strengthen activation of TGF-β signaling [25], which results in increased apoptosis and malalignment of vascular smooth muscle cells, ultimately increasing the risk of AD [6].

In addition, many studies have indicated that hypertension was risk factor for sporadic TAA, TAD, and abdominal aortic aneurysms (AAA) [55–61]; this is similar to the results of our study. Our study also observed that the risk of type B AD is higher for patients with a history of hypertension (Additional file 1: Table S1), which may be attributed to the mechanical affects of elevated blood pressure on the aortic wall [13]. Hypertension may bring about transform in medial SMCs and the ECM structural change, which effects the construction and function of the aortic wall, thereby increasing pressure on the aortic wall and boosting aortic dilatation [62]. Smoking was also associated with a higher risk of AD, which is consistent with the viewpoints of Landenhed M et al. [60]. The effect of smoking on aortic disease is mainly to change aortic SMCs and inflammatory response [51, 63]. Simultaneously, some mice studies have demonstrated that exposure to cigarette smoke result in AAA by inducing angiotensin II infusion or elastase perfusion [64, 65]. Consumption of ethanol may increase vasoconstriction by stimulating excitation of the sympathetic nervous system and secretion of norepinephrine, leading to vascular dysfunction and hypertension [66].

The early mortality of acute type B AD is more than 50%, if it is not treated in time [67]. Therefore, we further conducted a Kaplan–Meier curves showed that C carriers of rs145233125, G carriers of rs201170905 and G carriers of rs1800469 had a raised risk of death rate and recurring chest pain, which might be related to the continuous progression of type B AD. However, previous research evidence is still insufficient for the clinical outcomes of *FBN1*, *TGFB1* or *TGFB2* gene and patients with Stanford type B AD.

Certainly, the current research also exists some limitations. Firstly, since women suffer from AD is rare relatively than men, there may be gender differences. Secondly, the results of this study may be affected by different environmental factors. Thirdly, this was a single center study, which could not represent other population. A large sample and multi-center researches need to be conducted to further elucidation in future studies.

## Conclusions

In summary, *FBN1*rs201170905 and *TGFB1* rs1800469 genetic polymorphisms are related to the raised risks of type B AD. The interaction between *FBN1*, TGF-β signaling-related genetic polymorphisms, and environmental factors may promote the exacerbation of type B AD. G allele in rs201170905, C allele in rs145233125 of *FBN1*, G allele in *TGFB1* rs1800469 may be the prognostic indicators for type B AD in mortality and in chest pain recurrence.

## Supplementary Information

**Additional file 1: Table S1**. Logistic regression analysis of risk factors for type B AD.

## Data Availability

The data that support the findings of this study are available on request from the corresponding author. The data are not publicly available due to privacy or ethical restrictions.

## Abbreviations

AD: Aortic dissection
FBN1: Fibrillin-1
TGF-β: Transforming growth factor β
SNP: Single-nucleotide polymorphism
SMC: Smooth muscle cells
ECM: Extracellular matrix
EGF: Epidermal growth factor
MFS: Marfan syndrome
TAAD: Thoracic aortic aneurysm/dissection
TGFBR1: TGF-β type I receptor
GMDR: Generalized multifactor dimensionality reduction
TAD: Thoracic aortic dissection
TAA: Thoracic aortic aneurysm
AAA: Abdominal aortic aneurysms
