# Supplementary material for: Association of gene polymorphisms in FBN1 and TGF-β signaling with the susceptibility and prognostic outcomes of Stanford type B aortic dissection
Source: BMC Med Genomics. 2022 Mar 20;15:65. doi: 10.1186/s12920-022-01213-z (PMC8935688; doi:10.1186/s12920-022-01213-z)
Supplement: Supplementary file 1 — Additional file 1: Table S1. Logistic regression analysis of risk factors for type B AD. [file 12920_2022_1213_MOESM1_ESM.docx]

**Additional File:**

**Supplementary Table1 Logistic regression analysis of risk factors for type B AD.**

| **Characteristics** | **B** | **S.E.** | **Wald** | ***P*** | **OR** | **95CI** |
| --- | --- | --- | --- | --- | --- | --- |
| Age | -0.027 | 0.014 | 3.720 | 0.054 | 0.974 | 0.948-1.000 |
| Male | 0.303 | 0.346 | 0.768 | 0.381 | 1.354 | 0.687-2.669 |
| BMI | 0.018 | 0.035 | 0.264 | 0.607 | 1.018 | 0.951-1.090 |
| Triglyceride | -0.321 | 0.187 | 2.943 | 0.086 | 0.725 | 0.502-1.047 |
| Total cholesterol | 0.213 | 0.140 | 2.313 | 0.128 | 1.238 | 0.940-1.630 |
| Hypertension | 1.523 | 0.284 | 28.706 | 0.000 | 4.586 | 2.627-8.006 |
| Diabetes | -2.076 | 0.646 | 10.310 | 0.001 | 0.125 | 0.035-0.445 |
| Smoking | 0.728 | 0.360 | 4.087 | 0.043 | 2.071 | 1.022-4.194 |
| Drinking | 0.858 | 0.355 | 5.840 | 0.016 | 2.359 | 1.176-4.730 |
| constant | -4.486 | 1.405 | 10.196 | 0.001 | 0.011 |  |
